# Our Contemporaries

**Published:** 1912-04

**Authors:** 


### OUR CONTEMPORARIES

THE *Homeopathic Recorder* of January 1912, in its leading article, comments on Sir Henry Butlin's admission to the Royal College of Surgeons that the present probability is in favor of an intrinsic origin of cancer and asks why we should not hold the same view regarding poliomyelitis, tuberculosis, etc. We may reply that the reason is about the same which leads juries to indict certain men, bring in definite verdicts against others, and acquit still others. The tubercle bacillus has been tried and found guilty. Only the most unexpected discovery of fresh evidence can secure a reopening of the case. Poliomyelitis is in about the same state as a sudden death, with everything pointing toward murder and against suicide, with certain suspicions aroused but with no evidence sufficient to convict any individual. As to cancer, a number of arrests have been made, but in each case an alibi has apparently been proved, and many authorities are getting around to the opinion that it is a sort of suicide and not due to an extrinsic cause. The case is not clear as yet and we have no opinion to offer. But why should a disciple of Hahnemann who taught in the plainest way, even to the point of exciting ridicule, that diseases were due to an extrinsic, living cause, imply a hostility to the scientific working out of this theory?

---

The *Garden Magazine,* in its reading matter and advertisements, contains much matter of interest in regard to light farming, horticulture, poultry raising, care of animals, etc. It is a valuable adjunct in the cases of convalescence, mild invalidism, neurasthenia, overwork, incipient tuberculosis, etc., in which these occupations are of genuine therapeutic value either to prevent introspection, to afford exercise and out door life, or to add the means of earning a livlihood.

---

The *Milwaukee Medical Journal* has suspended publication, on account of the illness of the Editor, Dr. W. H. Neilson.

